# Genomic Insights into Bacterial Resistance to Proline-Rich Antimicrobial Peptide Bac7

**DOI:** 10.3390/membranes13040438

**Published:** 2023-04-17

**Authors:** Pavel V. Panteleev, Victoria N. Safronova, Roman N. Kruglikov, Ilia A. Bolosov, Tatiana V. Ovchinnikova

**Affiliations:** M.M. Shemyakin & Yu.A. Ovchinnikov Institute of Bioorganic Chemistry, The Russian Academy of Sciences, Miklukho-Maklaya Str., 16/10, 117997 Moscow, Russia; victoria.saf@ibch.ru (V.N.S.); kruglikov1911@mail.ru (R.N.K.); bolosov@ibch.ru (I.A.B.); ovch@ibch.ru (T.V.O.)

**Keywords:** antimicrobial peptide, cathelicidin, proline-rich peptide, Bac7, antibiotic, bacterial resistance, SbmA transporter, WaaP kinase, LPS

## Abstract

Proline-rich antimicrobial peptides (PrAMPs) having a potent antimicrobial activity and a modest toxicity toward mammalian cells attract much attention as new templates for the development of antibiotic drugs. However, a comprehensive understanding of mechanisms of bacterial resistance development to PrAMPs is necessary before their clinical application. In this study, development of the resistance to the proline-rich bovine cathelicidin Bac7_1-22_ derivative was characterized in the multidrug-resistant *Escherichia coli* clinical isolate causing the urinary tract infection. Three Bac7_1-22_-resistant strains with ≥16-fold increase in minimal inhibitory concentrations (MICs) were selected by serially passaging after four-week experimental evolution. It was shown that in salt-containing medium, the resistance was mediated by inactivation of the SbmA transporter. The absence of salt in the selection media affected both dynamics and main molecular targets under selective pressure: a point mutation leading to the amino acid substitution N159H in the WaaP kinase responsible for heptose I phosphorylation in the LPS structure was also found. This mutation led to a phenotype with a decreased susceptibility to both the Bac7_1-22_ and polymyxin B. Screening of antimicrobial activities with the use of a wide panel of known AMPs, including the human cathelicidin LL-37 and conventional antibiotics, against selected strains indicated no significant cross-resistance effects.

## 1. Introduction

Widespread antimicrobial resistance development is the cause of an escalating global health crisis, with multiple infectious diseases becoming increasingly difficult and expensive to treat. According to the World Health Organization, so-called ESKAPE (*Enterococcus faecium*, *Staphylococcus aureus*, *Klebsiella pneumoniae*, *Acinetobacter baumannii*, *Pseudomonas aeruginosa*, and *Enterobacter* spp.) pathogens are of particular concern. A further successful treatment of ESKAPE-mediated infections requires a novel pipeline for drug design to combat emerging resistance. Ideally, the selected candidate molecules must be free of pre-existing resistance.

Among new natural inhibitors of translation, animal proline-rich antimicrobial peptides (PrAMPs) are of special interest. PrAMPs form multiple interactions with the bacterial ribosome within the nascent peptide exit tunnel [1] that significantly reduce the risk of the bacterial resistance development due to modifications in ribosomal proteins or RNA and prevent cross-resistance in strains tolerant to small-molecule antibiotics with similar binding sites. On the other hand, a resistance to PrAMPs may arise as a result of mutations in transporter proteins, for example, in SbmA [2]. However, the obstacle can be overcome using combination of PrAMPs with pore-forming or membrane-targeting AMPs [3].

Among PrAMPs, the most studied one is bovine neutrophil cathelicidin Bac7 first described by Gennaro et al. more than 30 years ago [4]. Bac7 inhibits the elongation phase of the translation by sterically preventing the accommodation of the first aminoacyl-tRNA in the A-site of the ribosome [5,6]. Its shortened and modified analogues are of considerable interest as antibiotics against clinically significant Gram-negative pathogens [7]. It has been previously shown that the truncated *N*-terminal 22-23-residue fragments of Bac7 [8] and its structural homologs (the porcine PR-39 [9] and the caprine minibactenecin mini-ChBac7.5Nα [10]) retained the antibacterial activities of the wild-type PrAMPs. Recently, a 23-residue peptide Bac7PS, which demonstrated good efficacy in a murine septicemia model induced by *E. coli*, was obtained by a high-throughput screening of Bac7_1-23_ modified variants [11]. Despite a relatively low cytotoxicity compared to most other membranotropic antimicrobial peptides [12] and the lack of immunogenicity [13], a number of issues should be carefully considered before a possible implementation of PrAMPs in medical practice. Among them, the most important are (1) a probability of the appearance of phenotypically stable resistance mechanisms and the dynamics of its occurrence; (2) the cross-resistance to host-defense AMPs (cathelicidin LL-37, defensins, and others) and other molecular factors of the human immune system, as well as to antibiotics widely used in clinical practice; and (3) searching for possible ways to overcome mechanisms of potential resistance development.

In this work, we performed a comparative study of an experimental evolution of the bacterial resistance to Bac7_1-22_ fragment (RRIRPRPPRLPRPRPRPLPFPR) as a model well-studied PrAMP and to polymyxin B using the clinically isolated multidrug-resistant (MDR) *Escherichia coli* strain causing the urinary tract infection (UTI). Further whole-genome sequencing (WGS) analysis allowed us to identify two distinct mechanisms of the resistance development. Finally, potential fitness costs and cross-resistance effects were elucidated on a wide panel of known AMPs and conventional antibiotics.

## 2. Materials and Methods

### 2.1. Antimicrobial Agents

The chemically synthesized human cathelicidin LL-37, the *C*-terminally amidated 39-residue full-length VicBac, and melittin (>98% pure for all the peptides) were kindly provided by Dr. Maxim N. Zhmak and Dr. Sergey V. Sychev (M.M. Shemyakin and Yu.A. Ovchinnikov Institute of Bioorganic Chemistry of the Russian Academy of Sciences, Moscow, Russia). Other AMPs used in this study were produced in a bacterial expression system as described previously [3], including the recombinant analog of α-helical magainin-derived pexiganan, an antibacterial drug candidate [14]. Thioredoxin (Trx) was used as the fusion partner to ensure high yield of the peptide in the native conformation. The gene encoding pexiganan was obtained by annealing of two primers Pex-f and Pex-r (Table S1) followed by one-round DNA-polymerase extension and then cloned into pET-based vector as described previously [15]. The oligonucleotides used in this work were designed on the basis of *E. coli* K-12 codon usage bias. The target peptides were expressed in *E. coli* BL21 (DE3) as chimeric proteins that included 8×His tag, the *E. coli* thioredoxin A with the M37L substitution (TrxL), methionine residue, and a mature peptide. The cells transformed with the corresponding plasmid were grown at 37 °C in Lysogeny broth (LB) medium supplemented with 100 μg/mL ampicillin, 1 mM magnesium sulfate, and 20 mM glucose and were induced at OD_600_ 1.0 with 0.2 mM isopropyl β-D-1-thiogalactopyranoside (IPTG, Sigma, St. Louis, MO, USA) for 4 h at 30 °C and 220 rpm. After centrifugation, the pelleted cells were suspended and sonicated in the 100 mM phosphate buffer (pH 7.8) containing 20 mM imidazole and 6 M guanidine hydrochloride to fully solubilize the fusion protein. The clarified lysate was loaded on a column packed with Ni Sepharose (GE Healthcare, Chicago, IL, USA). The recombinant protein was eluted with the buffer containing 0.5 M imidazole. The eluate was acidified (up to pH 1.0) by the concentrated hydrochloric acid, and the fusion protein was cleaved by 100-fold molar excess of CNBr over methionine at 25 °C for 18 h in the dark. The lyophilized products of the cleavage reaction were dissolved in water and loaded on a semi-preparative Reprosil-pur C_18_-AQ column (10 × 250 mm, 5-μm particle size, Dr. Maisch GmbH, Ammerbuch-Entringen, Germany). Reversed-phase high-performance liquid chromatography (RP-HPLC) was performed with a linear gradient of acetonitrile in water containing 0.1% TFA (Figure S1A). The peaks were monitored at 214 and 280 nm, collected, and analyzed by MALDI-TOF MS using Reflex III mass-spectrometer (Bruker Daltonics, Bremen, Germany). All the obtained AMPs were analyzed by MALDI-TOF MS. The experimentally measured m/z values of the peptides matched well the corresponding calculated molecular masses. The obtained fraction with corresponding molecular mass (Table S2, Figure S1B) was dried in vacuo and dissolved in water. Polymyxin B and all used conventional antibiotics were purchased from Sigma (St. Louis, MO, USA).

### 2.2. Bacterial Strains

The clinical isolate *E. coli* MDR 1057 was collected and provided by the Sechenov First Moscow State Medical University hospital (Figure S2). The strain *E. coli* BW25113 and its knockout variants *E. coli* BW25113 *waaP*::kan (*E. coli* JW3605) and *E. coli* BW25113 *sbmA*::kan (*E. coli* JW0368) from the KEIO collection [16] were kindly provided by Dr. Petr V. Sergiev (Lomonosov Moscow State University, Russia).

### 2.3. Antimicrobial Assay

Bacterial test cultures were grown in the Mueller-Hinton broth (MHB, Sigma, St. Louis, MO, USA) at 37 °C to mid-log phase and then diluted with the 2× MHB media supplemented with 1.8% NaCl or without salt to reach a final cell concentration of 10^6^ CFU/mL. An amount of 50 µL of the obtained bacterial suspension was added to aliquots of 50 µL of the peptide solutions serially diluted with sterilized 0.1% bovine serum albumin (BSA) in 96-well flat-bottom polystyrene microplates (Eppendorf #0030730011, Hamburg, Germany). After incubation for 24 h at 37 °C and 950 rpm on the plate thermoshaker (Biosan, Riga, Latvia), minimum inhibitory concentrations (MIC) were determined as the lowest peptide concentrations that prevented growth of a test microorganism observed as visible turbidity. In most cases, no significant divergence of MIC values was observed (within ±1 dilution step). The results were expressed as the median values determined on the basis of at least three independent experiments performed in triplicate.

### 2.4. Resistance Induction Experiments

Resistance induction experiments were performed using the previously described method [3]. Briefly, on day one, the overnight culture of wild-type bacteria was diluted with the 2× MHB media supplemented with 1.8% NaCl to reach a final cell concentration of 10^6^ CFU/mL. An amount of 50 µL of the obtained bacterial suspension was added to aliquots of 50 µL of the peptide solutions serially diluted with the sterilized 0.1% BSA in 96-well flat-bottom polystyrene microplates. After incubation for 20 ± 2 h at 37 °C and 950 rpm, MICs were determined as described above. For each subsequent daily transfer, 2–4 μL of the inoculum taken from the first well containing a sub-inhibitory drug concentration was diluted with 2 mL of the fresh 2× MHB media supplemented with 1.8% NaCl. Then, 50 µL of this suspension was sub-cultured into the next passage wells containing 50 µL aliquots of the peptide at concentrations from 0.25× to 8–16× of the current MIC of each agent. Up to 25 repeated passages in the presence of antimicrobial agents were made for each bacterial strain during the experiment. Bacteria growing at the highest concentration of AMPs on the final day were passaged a further three times on drug-free agar plates before determining the final MIC value. Control serial passages in the absence of the agent were also included, and the resulting cultures showed unchanged MICs against antibacterial agents.

### 2.5. Whole-Genome Sequencing

To identify potential mechanisms conferring resistance to Bac7_1-22_ and polymyxin B, we performed a whole genome sequencing of resistant strain followed by genomic DNA de novo assembly and variant calling. The assembled wild type *E. coli* MDR 1057 strain genome was used as reference. Moreover, 2 × 100 bp pair-end sequencing of prepared genomic DNA was performed with an Illumina MiSeq platform (Illumina, San Diego, CA, USA). Evaluation of reads quality was performed using FastQC software (v0.11.9) [17], then reads were filtered and adapters were cut with TrimmomaticPE (v0.39) [18]. SPAdes software (v3.13.0) was used to assemble genomes utilizing both filtered paired-end and unpaired reads [19]. Assembly quality was then evaluated with QUAST program (v5.0.2) [20]. Gene prediction and annotation of assembled contigs was performed with Prokka program (v1.14.6) [21]. Alignment of paired-end reads on reference genome was made using BWA-MEM (v0.7.17-r1188) algorithm [22]. To call actual variants, VarScan software (v2.4.0) was launched with minimal reported variant frequency set to 0.9 [23].

### 2.6. Analysis of the sbmA Gene

The *sbmA* gene encoding the *E. coli* inner membrane transporter was amplified by polymerase chain reaction (PCR) using specific primers (Table S1). Individual bacterial colonies of the tested Bac7_1-22_-resistant strains were picked up from Petri dish and used as a template for PCR. The following components were mixed for the PCR: 2 µL of 10× Encyclo buffer (Evrogen, Moscow, Russia), 0.4 µL of 50× Encyclo DNA polymerase, 0.2 mM dNTPs, 10 µM Sbm-f primer, 10 µM Sbm-r primer, bacterial cells on inoculation loop, and water diluting to the total volume of 20 µL. Amplification was carried out on a thermocycler using initial denaturation (95 °C, 10 min), 25 amplification cycles (94 °C, 30 s; 55 °C, 40 s; 72 °C, 3 min), and final elongation (72 °C, 10 min). The products were separated by electrophoresis on 1.5% agarose gel (4 V/cm) and visualized on a UV trans-illuminator. The PCR products were purified from agarose gel and inserted into pAL2-T vector (Evrogen, Moscow, Russia). The ligation products were transformed into the chemically competent *E. coli* DH10B cells. Plasmid DNA was isolated from overnight cultures of single white colonies on LB agar plates supplemented with ampicillin (100 µg/mL), using Plasmid Miniprep kit (Evrogen, Moscow, Russia). The plasmids were sequenced on both strands using the ABI PRISM 3100-Avant automatic sequencer (Applied Biosystems, Foster City, CA, USA). At least two independent experiments were performed with each strain to prove the obtained results.

### 2.7. Construction of Complementation Plasmids

The plasmid pBAD/Myc-His A kindly provided by Dr. Olga Melkina (NRC “Kurchatov Institute”, Moscow, Russia) was used for the construction of the complementation plasmids overexpressing wild-type *waaP* gene from *E. coli* MDR 1057 and its mutant variant in WaaP-deficient *E. coli* BW25113 strain. Individual bacterial colonies were used as a template for PCR. Amplification of *waaP* gene was carried out using Waa-f and Waa-r primers (Table S1) and the following conditions: initial denaturation (95 °C, 10 min), 25 amplification cycles (94 °C, 30 s; 57 °C, 40 s; 72 °C, 60 s), and final elongation (72 °C, 10 min). The products were purified by electrophoresis on 1.5% agarose gel and cloned into pBAD/Myc-His A vector using NcoI/EcoRI restriction sites. The ligation products were transformed into the chemically competent *E. coli* DH10B cells. The obtained purified plasmids were sequenced and transformed into the chemically competent *E. coli* BW25113 *waaP*::kan strain. The antibacterial activity of Bac7_1-22_ against obtained strains was tested as described in Section 2.3. with the use of the salt-containing MHB media supplemented with ampicillin (100 µg/mL) and L-arabinose (0.1 g/L).

### 2.8. Growth Rate Determination

The overnight bacterial culture in LB was diluted with the same media to the 10^6^ CFU/mL, and 100 μL was added to 96-well flat-bottom polystyrene microplates to grow in 8 biological replicates at 37 °C in plate shaker (900 rpm). The OD at 570 nm was registered for 24 h with a AF2200 microplate reader (Eppendorf, Hamburg, Germany), and sterile LB medium was used as blank control.

### 2.9. Biofilm Assay

The *E. coli* MDR 1057 cells were incubated in LB medium for 16 h at 37 °C and then were diluted 150-fold with the same medium supplemented with 25 mM glucose or with the M9 minimal medium supplemented with 25 mM glucose, 5 µM thiamine, 1 mM MgSO_4_, 0.5 mM CaCl_2_, and the trace metals mixture. An amount of 100 µL of the obtained bacterial suspension was added to 96-well flat-bottom polystyrene microplates and incubated at 32 °C with gentle agitation (120 rpm) for 24 h to allow biofilm formation. Next, the medium was removed, wells were rinsed with sterile water three times, and 0.1% aqueous solution of crystal violet (CV, Sigma, St. Louis, MO, USA) was added for 40 min at 25 °C. Then, the liquid was removed, and wells were washed with sterile water to clean up the excess dye and 30% acetic acid was added to each well and incubated at 25 °C for 15 min for the extraction of CV from biofilms. Furthermore, the extracts were transferred to a new plate and absorption was measured at 570 nm with a AF2200 microplate reader. This experiment was carried out in triplicate. The data were analyzed by unpaired *t*-test using GraphPad Prism v.8.0.1.

## 3. Results and Discussion

### 3.1. Induction of Bacterial Resistance 

Most of the peptides studied in this work (except melittin, LL-37, and VicBac) were obtained using the same protocol of heterologous expression in bacteria with negligible deviations (Table S2). The selection of resistant strains makes it possible to elucidate both the key targets of AMPs and mechanisms of the resistance development. Here, we used the MDR uropathogenic *E. coli* 1057 strain isolated from a patient with UTI and sequenced in our previous study [15]. This strain bears the plasmid-mediated acquired resistance to beta-lactams (extended spectrum beta-lactamase (ESBL) producer, blaCTX-M-15), macrolides, and sulfamethoxazole (Figure S2). It also carries two well-characterized chromosomal mutations in *gyrA* (S83L and D87N), causing a high-level fluoroquinolone resistance as well as a higher-than-normal spontaneous mutation rate [24]. Thus, it is an applicable strain for laboratory modeling of bacterial evolution and development of resistance to drugs candidates appropriate for treatment of the infections caused by *E. coli* with multiple drug resistance.

**Figure 1:**
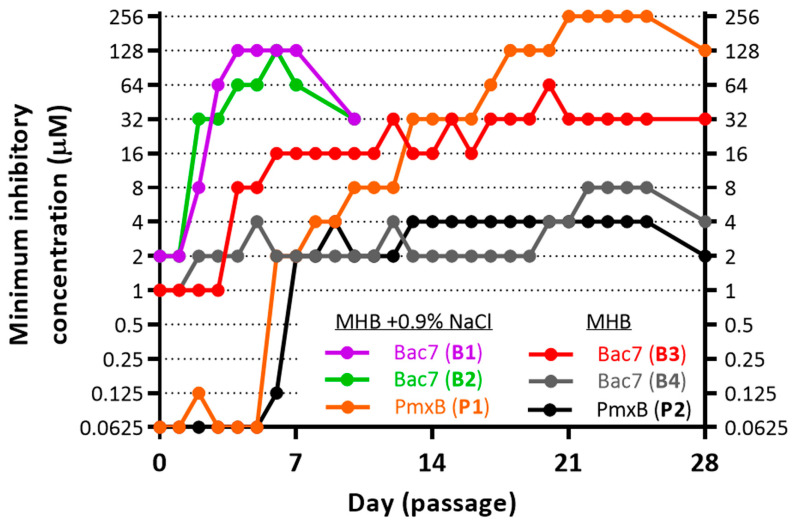
Serial passage induction of the resistance to Bac7_1-22_ and polymyxin B (PmxB) against the *E. coli* MDR 1057 strain in MHB media ±0.9% NaCl (salt). Bacteria growing at the highest concentration of AMPs after the final passage (on the 7th or 25th day) were further passaged three times on drug-free agar plates before determining the final MIC value.

Here, we performed serial passage induction of the resistance to Bac7_1-22_ and polymyxin B (PmxB) against the *E. coli* MDR 1057 strain in MHB media ±0.9% NaCl (salt). Antibacterial activities of some proline-rich AMPs were low when tested in the presence of 0.9% NaCl, which might inhibit absorption of the peptides to the bacterial surface [3]. The method used in this study allows monitoring MIC values after each transfer during long-term exposure to antimicrobials (Figure 1). As expected, no differences in MICs after 28 passages without antimicrobial agents were observed. The polymyxin B clinically used in treatment of the infections mediated by Gram-negative bacteria was chosen as a reference antibiotic in selection experiments. Here, we obtained two PmxB-resistant strains with final MIC values of 2 µM (32× MIC, the P2 strain) and 128 µM (2048× MIC, the P1 strain) (Figure 1). These data indicate that *E. coli* MDR 1057 strain can be used further as a model microorganism to achieve a high-level bacterial resistance to membrane-targeting antibiotics. A similar ≥1000-fold increase in MIC was also achieved when an extensively drug-resistant *Klebsiella pneumoniae* isolate was serially passaged in the presence of polymyxin B or colistin [25].

**Figure 2:**
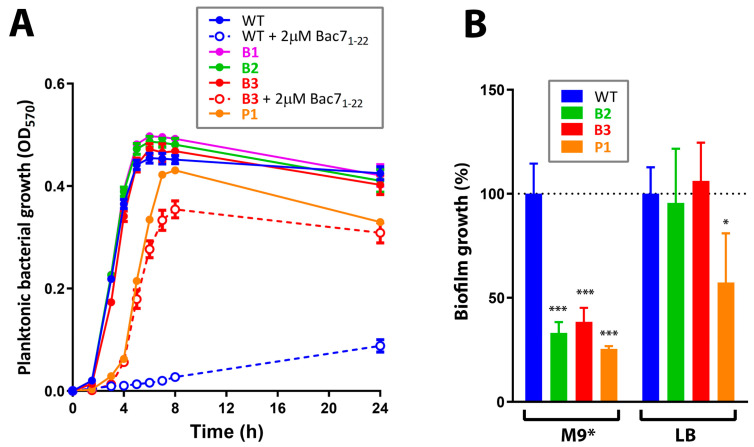
Fitness cost evaluation in selected strains. (**A**) Analysis of growth rates of the wild-type *E. coli* MDR 1057 (WT) and resistant strains (B1-B3, P1) in the LB medium. (**B**) Biofilm formation capacity of WT and resistant strains (B2, B3, P1) in the modified M9 medium or LB medium supplemented with glucose. Data are the mean ± SD of two independent experiments performed in triplicate. * *p* < 0.05, *** *p* < 0.001 significantly different compared to WT.

Similar to our previous experiments with other short PrAMPs such as VicBac_1-22_ and a panel of mini-ChBac7.5Nα analogs [3,15,26], ≥32-fold increases in MIC values were registered just after initial 3-4 passages subjected to selection by Bac7_1-22_. The obtained Bac7_1-22_-resistant strains were named as B1 and B2. Interestingly, the absence of 0.9% NaCl in the growth media greatly affected the profile of the resistance induction: in two parallel experiments (Figure 1), we found only 4- (strain B4) and 32-fold (strain B3) increase in MICs for Bac7_1-22_ after 4 weeks of selection. All of the three Bac7_1-22_-resistant strains (B1–B3) showed similar growth rates in the rich LB medium (Figure 2A) that pointed at insufficient fitness costs of acquired mutations.

Unlike Bac7_1-22_-resistant strains, a decreased planktonic growth kinetics was observed for the P1 strain. Importantly, the super-resistance to PmxB did not result in a biofilm-producing phenotype, which might also lead to chronic infections (Figure 2B). On the contrary, a significant decrease in the level of biofilm formation was found. The Bac7_1-22_-resistant strains B1-B3 and the PmxB-resistant strain P1 were then analyzed by WGS to identify possible genome mutations.

### 3.2. Resistance to Bac7_1-22_ in the Salt-Containing Medium Is Mediated by Inactivation of the SbmA Transporter 

**Figure 3:**
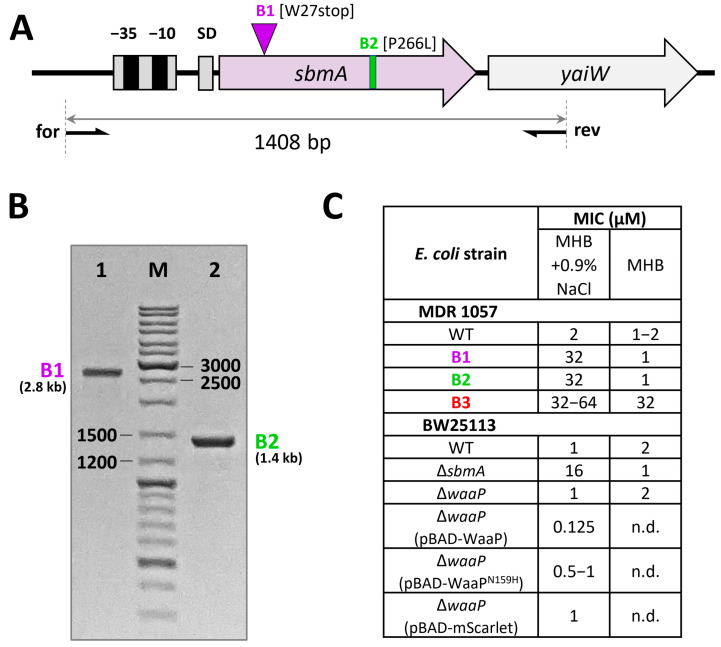
Analysis of the *sbmA* gene in Bac7_1-22_-resistant strains. (**A**) PCR-amplification scheme of the *sbmA-yaiW* gene region. The *yaiW* gene, located downstream of *sbmA*, is a part of the same operon. (**B**) Agarose gel analysis of the PCR-products after amplification of the *sbmA* gene. Lane 1—the strain B1; M—DNA molecular size marker; lane 2—the strain B2. (**C**) Antibacterial activity of Bac7_1-22_ against different *E. coli* strains in MHB media ±0.9% NaCl (salt). n.d., not determined.

In this study, using the Mueller-Hinton broth supplemented with 0.9% NaCl as the selection medium, we identified novel variants of inactivation of the *sbmA* gene (Table 1) mediated by (i) 1.4 kb insertion causing the premature stop codon [W27stop] (the strain B1) or (ii) a single nucleotide mutation causing [P266L] substitution (the strain B2). The genetic changes in both B1 and B2 strains were proved by PCR-amplification of the *sbmA* gene (Figure 3A,B) followed by cloning in plasmid vector and Sanger sequencing. In particular, a 2.8 kb band (rather than 1.4 kb) was found in the case of the strain B1 (Figure 3B).

PrAMPs primarily kill bacteria using a non-lytic mechanism without significantly affecting membrane integrity. Therefore, mutations leading to resistance to PrAMPs usually arise as a result of inactivation of specific transporter proteins SbmA/BacA family [27] widespread in *Proteobacteria* and *Actinobacteria* and/or due to loss of MdtM function. MdtM is an efflux pump that extrudes antibiotics from the bacterial cytosol [28]. SbmA is a homodimer proton-driven transporter found among some classes of *Proteobacteria* [2], which is involved in uptake of non-ribosomal peptide antibiotics [29], ribosomally synthesized and post-translationally modified peptides [30], and PrAMPs [1]. BacA in *Sinorhizobium meliloti* is important for establishing effective symbiosis with leguminous plants [31].

The SbmA transporter is a mutation-prone protein undergoing a strong selective pressure when short PrAMPs are used to induce bacterial resistance in vitro. Previously, different ways of the SbmA inactivation (insertions, deletions, single nucleotide mutations) have already been observed in salt-containing media for a number of mammalian PrAMPs and their analogs [3,15,26,32]. In another study, a 600 bp insertion (producing a stop codon) was identified in the *sbmA* gene after serial passages of the *E. coli* BL21AI strain in the diluted TSB medium in the presence of known insect PrAMP apidaecin 1b [33]. It is interesting to note that the sensitivity of selected strains B1 and B2 to Bac7_1-22_ was completely restored in the salt-free MHB medium (Figure 3C). Similar effects were shown in our previous studies with the use of various *E. coli* strains with the inactivated SbmA [3,15] including the knockout variant BW25113 *sbmA*::kan (Figure 3C). This pointed to a secondary role of SbmA in the transport of PrAMPs and a low selective pressure on the *sbmA* gene in a salt-free medium such as MHB. The absence of *sbmA* mutations in the strain B3, which was selected in a salt-free MHB medium (see Section 3.3), confirmed this finding. While the resistance to antibiotics and AMPs is often associated with a negative effect on fitness, the *sbmA* mutants of *S. enterica* were neither in vitro nor in vivo negatively affected in growth compared to the wild-type parental strain [32]. Similarly, we did not find a difference in the growth rate of the strains B1 and B2 as compared to the WT *E. coli* MDR 1057 strain (Figure 2A).

The possible ways to overcome the SbmA-mediated resistance are the design of more hydrophobic PrAMP analogs independent of specific transporters [7,15] or the use of synergy effects with endogenous AMPs expressed by the host (such as LL-37 in humans or CRAMP in mice) and facilitating intracellular transport of PrAMPs [34]. An alternative strategy is the use of PrAMPs for the treatment of specific pathogens for which the loss of specific transporters decreases the virulence. For example, *Mycobacteria* seem to be a rare group of Gram-positive species that harbor a BacA-like ABC transporter which similarly to SbmA mediates the uptake of PrAMPs such as Bac7 and non-ribosomal peptide antibiotics [29]. In *Mycobacterium tuberculosis*, this protein (also known as Rv1819c) is essential for the import of vitamin B12 [35,36] and needed for the maintenance of chronic infection in mice [37]. In contrast to SbmA from *Enterobacteriaceae* species, the inactivation of Rv1819c significantly reduced the virulence of *M. tuberculosis* in mice [37]. Additionally, the BacA-deficient *M. tuberculosis* also became sensitive to the human defensin HBD2 relative to the corresponding wild-type strain [38]. In total, this makes PrAMPs promising antituberculotic agents. Thus, PR-39 was shown to inhibit growth of *M. tuberculosis* H37Rv at a concentration of about 10 µM [39]. In our previous work, we also found the novel ribosome-targeting proline-rich cathelicidin VicBac from alpaca effectively killing fast-growing *Mycobacterium phlei* [15] and *Mycobacterium smegmatis* mc(2)155 (unpublished data) with the MIC values of 0.5–1 µM.

### 3.3. The Single Mutation in the WaaP Kinase Contributes to E. coli Resistance to Bac7_1-22_

**Figure 4:**
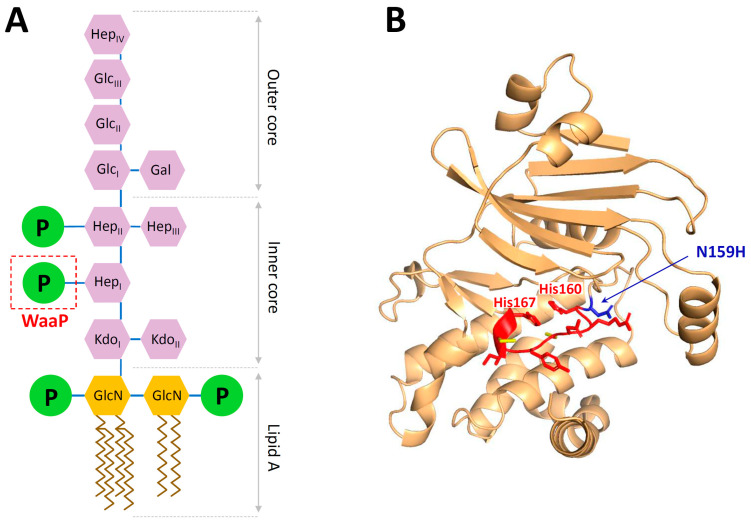
Analysis of the *waaP* gene mutation in the Bac7_1-22_-resistant strain B3. (**A**) Schematic representation of the structure of the core oligosaccharide and lipid A of LPS of *E. coli* based on [45]. The site of the WaaP kinase action is highlighted in the red dashed box. GlcN, glucosamine; KDO, 2-keto-3-deoxyoctulosonic acid; Hep, L-glycero-D-manno-heptose; Gal, galactose; Glc, glucose; P, phosphate. (**B**) Spatial structure of the WaaP kinase from the *E. coli* MDR 1057 strain predicted by AlphaFold2 program [46]. The WaaP conservative catalytic motif is colored with red. The substitution N159H found in the strain B3 is colored with blue.

The whole-genome sequencing of the strain B3 with lower sensitivity to Bac7_1-22_ (MIC of 64 and 32 µM in MHB with or without 0.9% NaCl, respectively) showed two changes in the genome: the point mutation in the *waaP* gene (also referred as *rfaP*) causing the amino acid substitution Asn159His, and the point mutation in the intergenic region between *menE* and *pmrD* genes (Table 1). In *E. coli*, the *waa* gene cluster is involved in LPS core biosynthesis and modification, whose structure is critical for outer membrane stability (Figure 4A). The kinase is required for phosphorylation of the inner heptose in the core oligosaccharide, on which depend the modifications of subsequent heptose [40]. So far, the mutations in *waa* genes associated with resistance to PrAMPs were described only for PR-39 (*waaY* and *waaG*) [41]. In this study, we found a mutation in *waaP* leading to a pronounced resistance to Bac7_1-22_. Being a cytoplasmic enzyme, WaaP unlikely interacts with Bac7, since the 70S ribosome and the chaperone DnaK have previously been shown as two main intracellular targets of the peptide [1]. The bacterial WaaP shares a common mechanism of action with eukaryotic protein kinases [40,42]. In *E. coli*, a highly conserved catalytic motif of WaaP (HRDCYICH) is located between His160 and His167 residues that suggests a possible inactivation of this enzyme due to the mutation of Asn159 near this site (Figure 4B). The inactivation of *waaP* leads to significant structural changes of LPS, associated with the deep-rough phenotype and hypersensitivity to hydrophobic antibiotics in some *E. coli* strains [43], and characterized by a loss of the *S. enterica* virulence in vivo [44]. Here, we did not find any significant difference in the sensitivity of WT and B3 strains to quite hydrophobic AMPs such as LL-37 and protegrin-1 (Table 2). In contrast, a cross-resistance to some AMPs and polymyxin B was detected, which is discussed in Section 3.5.

Next, we checked the susceptibility of the knockout variant *E. coli* BW25113 *waaP*::kan strains complemented with a plasmid expressing either the wild-type *waaP* or the mutant *waaP*^N159H^ (Figure 3C). The MIC values of Bac7_1-22_ against both *E. coli* BW25113 and its Δ*waaP* variant were found to be similar (1 µM). Notably, the pBAD plasmid-mediated overexpression of the WaaP kinase in the *waaP*-knockout strain was found to be toxic to the host and resulted in complete inhibition of cell growth at arabinose concentration above 1 g/L. The observed toxic effect was similar for both wild-type and the mutant N159H that pointed at the biological activity of both kinase variants. The overexpression of the control plasmid pBAD-mScarlet (RFP-derived fluorescent protein with the size similar to that of WaaP) did not alter cell growth even at arabinose concentration above 10 g/L. Therefore, we used 0.1 g/L arabinose for the expression of target genes in the following complementation experiments. As the result, we found 4–8-fold increase in MICs for the strain expressing *waaP*^N159H^ (MIC of 0.5–1 µM) as compared with the wild-type variant (MIC of 0.125 µM) that proved a possible role of this mutation in adaptation to Bac7 (Figure 3C). Expectedly, the MIC value of Bac7_1-22_ against the *waaP*-knockout strain expressing pBAD-mScarlet was of 1 µM. In sum, we assumed that the found mutation N159H apparently modulated the activity of the WaaP kinase rather than inactivated it. This is a preliminary result, and we also cannot exclude different levels of the *waaP* expression in the complementation experiments. For an in-depth study of the effect of this mutation, it is planned further to analyze the LPS structure of the B3 strain and analyze an activity of the mutant WaaP variant as well as to introduce the *waaP* mutation into a clean background *E. coli*. We also cannot exclude that a mutation in the *menE*→*pmrD* intergenic region can alter a bacterial sensitivity to Bac7_1-22_ (Figure S4). It was shown that PmrD in *E. coli* influenced the expression of *pmrA* and its downstream targets, including the genes encoding the lipid A modification enzymes [47]. It should be noted that the treatment of septic shock-modelled rats with Bac7_1-35_ resulted in a significant decrease in plasma LPS levels and lethality rates comparable with that for polymyxin B [48]. In sum, our findings indicated that specific interactions with the LPS structure might be an important step in the translocation of Bac7 inside *E. coli* cells.

### 3.4. The Super-Resistance of E. coli to Polymyxin B Is Mediated by Seven Genome Mutations

An adaptive resistance of ESKAPE pathogens to polymyxin B, a drug of last resort, is also an emerging problem worldwide [49]. The resistance to polymyxins is typically achieved via cell envelope modifications that reduce affinity to these lipopeptides. These modifications often include the decoration of the lipid A component of lipopolysaccharide with 4-amino-4-deoxy-L-arabinose (L-Ara4N) and/or phosphoethanolamine (pEtN), which, respectively, reduce or redistribute negative charge of the cell envelope and subsequently lower affinity to positively charged polymyxins resulted to MICs of about 4–8 µM [49]. Two of seven found mutations in the strain P1 are located in the gene encoding PmrB (Table 1). PmrB is a sensor histidine kinase which has been shown to play an important role in the regulation of the lipid A modification system [50,51]. It is interesting to note that both mutations were previously described in the *E. coli* strain isolated from swine feces [52] and also from the resistant *Salmonella enterica* strain which was in vitro selected by plating on agar medium supplemented with colistin [53]. Each of them independently led to an increase in MIC of PmxB/colistin up to 3 µg/mL (4 µM) [53]. Here, in both repetitions (P1 and P2, Figure 1), an increase in MIC of polymyxin B from 0.0625 to 2 µM during the first week of the experiment was observed. It can be assumed that the rapid evolution of the resistance to polymyxins was mainly contributed by mutations in PmrB.

Interestingly, other mutations found in P1 (*bamA*^I130S^, *sppA*^R371H^, *spoT*^N118H^, *rpoS*^Q304stop^, *secA*^Q830stop^) have not previously been described in the context of the polymyxin resistance. The interactions with atypical protein targets localized in the periplasmic space or outer membrane (for example, SppA and BamA) may be assumed when polymyxin is administrated at extremely high concentrations. For example, the ability of the murepavadin-polymyxin chimeric compound to bind to the outer membrane protein BamA was shown [54]. On the other hand, the above-mentioned proteins (SecA, BamA, SpoT) are directly or indirectly involved in the biogenesis of bacterial membranes, and mutations in these proteins necessitate changes in the cell surface. A compensatory origin of any of these mutations also cannot be excluded.

### 3.5. Screening of Cross-Resistance Effects

Finally, we explored whether the laboratory evolved resistance to Bac7_1-22_ (strains B2 and B3) and to PmxB (P1) provide any cross-resistance to 10 conventional clinically used antibiotics or other known and well-studied cationic AMPs. In particular, we measured the changes in susceptibilities of the strains to (i) membrane-targeting linear α-helical AMPs (melittin from the bee venom, the caprine cathelicidin ChMAP-28 [3], the human cathelicidin LL-37, and pexiganan—a broad spectrum magainin-derived peptide for the treatment of diabetic foot ulcer infections [14]); (ii) β-hairpin AMPs (the LptA-targeting insect thanatin [55], the membrane-targeting porcine cathelicidin protegrin-1, the horseshoe crab tachyplesin-1, and AA139—the arenicin-derived lead candidate for the treatment of Gram-negative MDR infections [56]); and (iii) linear proline-rich ribosome-targeting cathelicidins PR-39_1-22_, mini-ChBac7.5Nα [3], and VicBac [15]. The activity of the obtained recombinant pexiganan was similar to that of the synthetic one according to [14] (Table 2). Cross-resistance was defined as a minimum of 4-fold increase in the resistance level (Table 2).

Expectedly, a pronounced cross-effect of Bac7_1-22_-resistant strains B2 and B3 with other truncated PrAMPs with a similar mechanism of action was found. It is important to note that the use of PrAMP with the *C*-terminal hydrophobic motif (for example, VicBac) allowed to successfully overcome both SbmA- and WaaP-mediated resistance (Table 2). A pronounced cross-resistance to polymyxin and thanatin, as well as a moderate effect against AA139, was found for the strain B3. Notably, a specific binding to LPS is an important step in the realization of the antimicrobial action by these peptides. We also found the cross-resistance of the PmxB-resistant strain P1 to the representatives of all structural classes of tested AMPs with the most pronounced effects against PrAMPs. In total, this indicated significant changes in the cell envelope of P1 compared to WT strain. Fortunately, we did not find any cross-effects in B2, B3, and P1 towards all tested conventional antibiotics and LL-37. The last one is known as a key host-defense cathelicidin presented throughout the human body.

## 4. Conclusions

AMP-based molecules are promising novel antimicrobial agents, but evolution of resistance towards these agents is a serious health concern. In this study, we performed a comparative study of the experimental bacterial resistance to the Bac7_1-22_ fragment served as a model of a typical ribosome-targeting PrAMP and to polymyxin B as a reference drug, using the clinically isolated MDR *Escherichia coli* strain causing the urinary tract infection. The experiment revealed that the presence of salt in the medium drove the evolution of the Bac7 resistance to the simplest path, namely an inactivation of the non-essential SbmA transporter, that is accompanied by insufficient fitness costs. The role of SbmA in the peptide transport is likely minimized in a medium with a low ionic strength; therefore, other genes mutate under the selection. For example, the appearance of a mutation in the WaaP kinase in the Bac7-resistant strain was shown for the first time, which indicates a potential role of the LPS structure in the peptide translocation inside the target bacterial cell. The strain with the mutant WaaP kinase exhibited cross-resistance to other short PrAMPs, as well as to a number of AMPs (polymyxin B, thanatin, AA139, but not tachyplesin-1). Mechanisms of action of these peptides are associated with their interaction with the bacterial LPS. The results obtained also indicated a pronounced ability of the MDR uropathogenic *E. coli* 1057 strain to generate a high-level resistance to AMPs that was demonstrated with the PmxB-resistant strain P1 harboring seven genome mutations which resulted in a stable phenotype with the MIC value of >128 µM.

The lack of growth defects in Bac7-resistant *E. coli* strains harboring mutations in *sbmA* or *waaP* genes is a quite worrying finding since it suggests that AMP-resistant mutants might have fitness similar to that of a susceptible strain when growing in a host. On the other hand, we did not find any significant cross-effects towards the human key host-defense cathelicidin LL-37 and all tested clinically relevant antibiotics except for polymyxin B. Despite the acquired bacterial resistance to Bac7_1-22_ may arise in vivo, the risk of compromising innate immunity factors and reducing the effectiveness of standard antibiotic therapy appears to be negligible. We also believe that the design of transporter-independent analogs of natural peptides or their application against specific pathogens such as *Mycobacteria* will facilitate the development of PrAMP-based ribosome-targeting antibacterial drugs in the near future.

## Figures and Tables

**Table 1:** Mutations identified in *E. coli* strains with resistance to Bac7_1-22_ and polymyxin B.

Strain	Selection Conditions	Mutation	Type	Gene	Gene Product
B1	MHB + 0.9%NaCl Bac7_1-22_ (7 passages)	W27stop (TG**G**→TG**A**)	insertion (IC3-like element, 1.4 kb)	*sbmA*	cytoplasmic membrane transporter
B2	MHB + 0.9%NaCl Bac7_1-22_ (7 passages)	P266L(C**C**G→C**T**G)	single nucleotide polymorphism	*sbmA*	cytoplasmic membrane transporter
B3	MHB Bac7_1-22_ (25 passages)	N159H (**A**AC→**C**AC)	single nucleotide polymorphism	*waaP*	heptose specific lipopolysaccharide (LPS) core kinase
		21st position (G→T)	single nucleotide polymorphism	*menE*/ *pmrD*	intergenic 109 bp region
P1	MHB + 0.9%NaCl PmxB (25 passages)	I130S (A**T**T→A**G**T)	single nucleotide polymorphism	*bamA*	outer membrane protein assembly complex
		R371H (C**G**T→C**A**T)	single nucleotide polymorphism	*sppA*	cytoplasmic membrane signal peptide peptidase
		N118H (**A**AC→**C**AC)	single nucleotide polymorphism	*spoT*	bifunctional (p)ppGpp synthase/hydrolase
		Q304stop (**C**AG→**T**AG)	in-frame stop codon	*rpoS*	RNA polymerase sigma factor σ^S^
		Q830stop (**C**AG→**T**AG)	in-frame stop codon	*secA*	protein translocation ATPase
		V161G (G**T**G→G**G**G)	single nucleotide polymorphism	*pmrB (basS)*	cytoplasmic membrane sensor histidine kinase PmrB
		S305R (**A**GT→**C**GT)	single nucleotide polymorphism		

**Table 2:** Analysis of cross-resistance of B2, B3, and P1 to AMPs and conventional antibiotics.

Antibacterial Agent			Minimum Inhibitory Concentration, µM			
			WT	B2	B3	P1
Antimicrobial peptides	proline-rich	Bac7_1-22_	2	>32	>32	16 *
		mini-ChBac7.5Nα	8	>32	>32	>32
		PR-39_1-22_	2	>32	>32	16
		VicBac	1	2	2	4
	β-hairpin	AA139 **	0.25	0.25	1	0.5
		Tachyplesin-1	0.063	0.063	0.063	0.063
		Protegrin-1	0.25	0.25	0.25	0.25
		Thanatin	1	1	>32	>32
	α-helical	Pexiganan **	1	1	2	8
		LL-37	4	2	2	1
		ChMAP-28	0.063	0.063	0.063	0.063
		Melittin	8	8	4	4
	Polymyxin B		0.125	0.125	8	>128
Conventional antibiotics	Ampicillin		256	256	256	256
	Ceftriaxone		128	128	128	128
	Ciprofloxacin		256	128	256	128
	Rifampicin		64	128	64	64
	Tetracycline		8	16	8	8
	Gentamicin		2	2	2	4
	Chloramphenicol		8	16	8	16
	Spectinomycin		32	32	32	64
	Clindamycin		128	256	128	256
	Roxithromycin		256	256	256	>256

* ≥4-fold increase in MIC as compared to WT was classified as cross-resistance effect (marked with red). ** AMP-based antibiotics under preclinical or clinical trials.

## Data Availability

All data generated and analyzed during this study are included in this published article and its “Supplementary Materials” file.

## Supplementary Materials

The following supporting information can be downloaded at: https://www.mdpi.com/article/10.3390/membranes13040438/s1, Figure S1: Purification of the recombinant pexiganan; Figure S2: Identification of acquired antibiotic resistance genes in *E. coli* MDR 1057 strain; Figure S3: pBAD-based plasmid vector for the expression of *waaP* under the tightly regulated arabinose promoter; Figure S4: A fragment of *E. coli* MDR 1057 genome sequence showing intergenic *menE-pmrD* region; Table S1: Oligonucleotide primers used in this study; Table S2: Amino acid sequences and molecular masses of AMPs used in this study. References [3,15,57,58,59,60] are cited in the supplementary materials.

## References

[1] Graf M., Mardirossian M., Nguyen F., Seefeldt A.C., Guichard G., Scocchi M., Innis C.A., Wilson D.N. (2017). Proline-Rich Antimicrobial Peptides Targeting Protein Synthesis. Nat. Prod. Rep..

[2] Ghilarov D., Inaba-Inoue S., Stepien P., Qu F., Michalczyk E., Pakosz Z., Nomura N., Ogasawara S., Walker G.C., Rebuffat S. (2021). Molecular Mechanism of SbmA, a Promiscuous Transporter Exploited by Antimicrobial Peptides. Sci. Adv..

[3] Panteleev P.V., Bolosov I.A., Kalashnikov A.À., Kokryakov V.N., Shamova O.V., Emelianova A.A., Balandin S.V., Ovchinnikova T.V. (2018). Combined Antibacterial Effects of Goat Cathelicidins with Different Mechanisms of Action. Front. Microbiol..

[4] Gennaro R., Skerlavaj B., Romeo D. (1989). Purification, Composition, and Activity of Two Bactenecins, Antibacterial Peptides of Bovine Neutrophils. Infect. Immun..

[5] Seefeldt A.C., Graf M., Pérébaskine N., Nguyen F., Arenz S., Mardirossian M., Scocchi M., Wilson D.N., Innis C.A. (2016). Structure of the Mammalian Antimicrobial Peptide Bac7(1–16) Bound within the Exit Tunnel of a Bacterial Ribosome. Nucleic Acids Res..

[6] Gagnon M.G., Roy R.N., Lomakin I.B., Florin T., Mankin A.S., Steitz T.A. (2016). Structures of Proline-Rich Peptides Bound to the Ribosome Reveal a Common Mechanism of Protein Synthesis Inhibition. Nucleic Acids Res..

[7] Mardirossian M., Sola R., Beckert B., Valencic E., Collis D.W.P., Borišek J., Armas F., Di Stasi A., Buchmann J., Syroegin E.A. (2020). Peptide Inhibitors of Bacterial Protein Synthesis with Broad Spectrum and SbmA-Independent Bactericidal Activity against Clinical Pathogens. J. Med. Chem..

[8] Benincasa M., Scocchi M., Podda E., Skerlavaj B., Dolzani L., Gennaro R. (2004). Antimicrobial Activity of Bac7 Fragments against Drug-Resistant Clinical Isolates. Peptides.

[9] Veldhuizen E.J.A., Schneider V.A.F., Agustiandari H., van Dijk A., Tjeerdsma-van Bokhoven J.L.M., Bikker F.J., Haagsman H.P. (2014). Antimicrobial and Immunomodulatory Activities of PR-39 Derived Peptides. PLoS ONE.

[10] Shamova O.V., Orlov D.S., Zharkova M.S., Balandin S.V., Yamschikova E.V., Knappe D., Hoffmann R., Kokryakov V.N., Ovchinnikova T.V. (2016). Minibactenecins ChBac7.Nα and ChBac7. Nβ—Antimicrobial Peptides from Leukocytes of the Goat Capra Hircus. Acta Nat..

[11] Koch P., Schmitt S., Heynisch A., Gumpinger A., Wüthrich I., Gysin M., Shcherbakov D., Hobbie S.N., Panke S., Held M. (2022). Optimization of the Antimicrobial Peptide Bac7 by Deep Mutational Scanning. BMC Biol..

[12] Tripathi A.K., Kumari T., Tandon A., Mohd S., Afshan T., Kathuria M., Shukla P.K., Mitra K., Ghosh J.K. (2017). Selective Phenylalanine to Proline Substitution for Improved Antimicrobial and Anticancer Activities of Peptides Designed on Phenylalanine Heptad Repeat. Acta Biomater..

[13] Holfeld L., Herth N., Singer D., Hoffmann R., Knappe D. (2015). Immunogenicity and Pharmacokinetics of Short, Proline-Rich Antimicrobial Peptides. Future Med. Chem..

[14] Lamb H.M., Wiseman L.R. (1998). Pexiganan Acetate. Drugs.

[15] Panteleev P.V., Safronova V.N., Kruglikov R.N., Bolosov I.A., Bogdanov I.V., Ovchinnikova T.V. (2022). A Novel Proline-Rich Cathelicidin from the Alpaca *Vicugna pacos* with Potency to Combat Antibiotic-Resistant Bacteria: Mechanism of Action and the Functional Role of the C-Terminal Region. Membranes.

[16] Baba T., Ara T., Hasegawa M., Takai Y., Okumura Y., Baba M., Datsenko K.A., Tomita M., Wanner B.L., Mori H. (2006). Construction of *Escherichia coli* K-12 In-frame, Single-gene Knockout Mutants: The Keio Collection. Mol. Syst. Biol..

[17] de Sena Brandine G., Smith A.D. (2021). Falco: High-Speed FastQC Emulation for Quality Control of Sequencing Data. F1000Research.

[18] Bolger A.M., Lohse M., Usadel B. (2014). Trimmomatic: A Flexible Trimmer for Illumina Sequence Data. Bioinformatics.

[19] Bankevich A., Nurk S., Antipov D., Gurevich A.A., Dvorkin M., Kulikov A.S., Lesin V.M., Nikolenko S.I., Pham S., Prjibelski A.D. (2012). SPAdes: A New Genome Assembly Algorithm and Its Applications to Single-Cell Sequencing. J. Comput. Biol..

[20] Gurevich A., Saveliev V., Vyahhi N., Tesler G. (2013). QUAST: Quality Assessment Tool for Genome Assemblies. Bioinformatics.

[21] Seemann T. (2014). Prokka: Rapid Prokaryotic Genome Annotation. Bioinformatics.

[22] Li H., Durbin R. (2009). Fast and Accurate Short Read Alignment with Burrows-Wheeler Transform. Bioinformatics.

[23] Koboldt D.C., Zhang Q., Larson D.E., Shen D., McLellan M.D., Lin L., Miller C.A., Mardis E.R., Ding L., Wilson R.K. (2012). VarScan 2: Somatic Mutation and Copy Number Alteration Discovery in Cancer by Exome Sequencing. Genome Res..

[24] Komp Lindgren P., Karlsson A., Hughes D. (2003). Mutation Rate and Evolution of Fluoroquinolone Resistance in *Escherichia coli* Isolates from Patients with Urinary Tract Infections. Antimicrob. Agents Chemother..

[25] Pitt M.E., Cao M.D., Butler M.S., Ramu S., Ganesamoorthy D., Blaskovich M.A.T., Coin L.J.M., Cooper M.A. (2019). Octapeptin C4 and Polymyxin Resistance Occur via Distinct Pathways in an Epidemic XDR *Klebsiella pneumoniae* ST258 Isolate. J. Antimicrob. Chemother..

[26] Bolosov I.A., Panteleev P.V., Balandin S.V., Shamova O.V., Ovchinnikova T.V. (2023). Structural and Functional Characteristics of the Proline-Rich Antimicrobial Peptide Minibactenecin from Leukocytes of Domestic Goat Capra Hircus. Bull. Exp. Biol. Med..

[27] Slotboom D.J., Ettema T.W., Nijland M., Thangaratnarajah C. (2020). Bacterial Multi-solute Transporters. FEBS Lett..

[28] Krizsan A., Knappe D., Hoffmann R. (2015). Influence of the *YjiL-MdtM* Gene Cluster on the Antibacterial Activity of Proline-Rich Antimicrobial Peptides Overcoming *Escherichia coli* Resistance Induced by the Missing SbmA Transporter System. Antimicrob. Agents Chemother..

[29] Imai Y., Hauk G., Quigley J., Liang L., Son S., Ghiglieri M., Gates M.F., Morrissette M., Shahsavari N., Niles S. (2022). Evybactin Is a DNA Gyrase Inhibitor That Selectively Kills *Mycobacterium tuberculosis*. Nat. Chem. Biol..

[30] Metelev M., Osterman I.A., Ghilarov D., Khabibullina N.F., Yakimov A., Shabalin K., Utkina I., Travin D.Y., Komarova E.S., Serebryakova M. (2017). Klebsazolicin Inhibits 70S Ribosome by Obstructing the Peptide Exit Tunnel. Nat. Chem. Biol..

[31] Glazebrook J., Ichige A., Walker G.C. (1993). A Rhizobium Meliloti Homolog of the *Escherichia coli* Peptide-Antibiotic Transport Protein SbmA Is Essential for Bacteroid Development. Genes Dev..

[32] Pränting M., Negrea A., Rhen M., Andersson D.I. (2008). Mechanism and Fitness Costs of PR-39 Resistance in *Salmonella enterica* Serovar Typhimurium LT2. Antimicrob. Agents Chemother..

[33] Schmidt R., Krizsan A., Volke D., Knappe D., Hoffmann R. (2016). Identification of New Resistance Mechanisms in *Escherichia coli* against Apidaecin 1b Using Quantitative Gel- and LC–MS-Based Proteomics. J. Proteome Res..

[34] Knappe D., Kabankov N., Herth N., Hoffmann R. (2016). Insect-Derived Short Proline-Rich and Murine Cathelicidin-Related Antimicrobial Peptides Act Synergistically on Gram-Negative Bacteria in Vitro. Future Med. Chem..

[35] Gopinath K., Venclovas C., Ioerger T.R., Sacchettini J.C., McKinney J.D., Mizrahi V., Warner D.F. (2013). A Vitamin B₁₂ Transporter in *Mycobacterium tuberculosis*. Open Biol..

[36] Rempel S., Gati C., Nijland M., Thangaratnarajah C., Karyolaimos A., de Gier J.W., Guskov A., Slotboom D.J. (2020). A Mycobacterial ABC Transporter Mediates the Uptake of Hydrophilic Compounds. Nature.

[37] Domenech P., Kobayashi H., LeVier K., Walker G.C., Barry C.E. (2009). BacA, an ABC Transporter Involved in Maintenance of Chronic Murine Infections with *Mycobacterium tuberculosis*. J. Bacteriol..

[38] Arnold M.F.F., Haag A.F., Capewell S., Boshoff H.I., James E.K., McDonald R., Mair I., Mitchell A.M., Kerscher B., Mitchell T.J. (2013). Partial Complementation of *Sinorhizobium meliloti* BacA Mutant Phenotypes by the *Mycobacterium tuberculosis* BacA Protein. J. Bacteriol..

[39] Linde C.M., Hoffner S.E., Refai E., Andersson M. (2001). In Vitro Activity of PR-39, a Proline-Arginine-Rich Peptide, against Susceptible and Multi-Drug-Resistant *Mycobacterium tuberculosis*. J. Antimicrob. Chemother..

[40] Yethon J.A., Whitfield C. (2001). Purification and Characterization of WaaP from *Escherichia coli*, a Lipopolysaccharide Kinase Essential for Outer Membrane Stability. J. Biol. Chem..

[41] Spohn R., Daruka L., Lázár V., Martins A., Vidovics F., Grézal G., Méhi O., Kintses B., Számel M., Jangir P.K. (2019). Integrated Evolutionary Analysis Reveals Antimicrobial Peptides with Limited Resistance. Nat. Commun..

[42] Kreamer N.N.K., Chopra R., Caughlan R.E., Fabbro D., Fang E., Gee P., Hunt I., Li M., Leon B.C., Muller L. (2018). Acylated-Acyl Carrier Protein Stabilizes the *Pseudomonas aeruginosa* WaaP Lipopolysaccharide Heptose Kinase. Sci. Rep..

[43] Yethon J.A., Heinrichs D.E., Monteiro M.A., Perry M.B., Whitfield C. (1998). Involvement of WaaY, WaaQ, and WaaP in the Modification of *Escherichia coli*Lipopolysaccharide and Their Role in the Formation of a Stable Outer Membrane. J. Biol. Chem..

[44] Yethon J.A., Gunn J.S., Ernst R.K., Miller S.I., Laroche L., Malo D., Whitfield C. (2000). *Salmonella enterica* Serovar Typhimurium WaaP Mutants Show Increased Susceptibility to Polymyxin and Loss of Virulence In Vivo. Infect. Immun..

[45] Wang Z., Wang J., Ren G., Li Y., Wang X. (2015). Influence of Core Oligosaccharide of Lipopolysaccharide to Outer Membrane Behavior of *Escherichia coli*. Mar. Drugs.

[46] Mirdita M., Schütze K., Moriwaki Y., Heo L., Ovchinnikov S., Steinegger M. (2022). ColabFold: Making Protein Folding Accessible to All. Nat. Methods.

[47] Rubin E.J., Herrera C.M., Crofts A.A., Trent M.S. (2015). PmrD Is Required for Modifications to *Escherichia coli* Endotoxin That Promote Antimicrobial Resistance. Antimicrob. Agents Chemother..

[48] Ghiselli R., Giacometti A., Cirioni O., Circo R., Mocchegiani F., Skerlavaj B., D’Amato G., Scalise G., Zanetti M., Saba V. (2003). Neutralization of Endotoxin In Vitro and In Vivo by BAC7(1-35), a Proline-Rich Antibacterial Peptide. Shock.

[49] Liu Y.-Y., Wang Y., Walsh T.R., Yi L.-X., Zhang R., Spencer J., Doi Y., Tian G., Dong B., Huang X. (2016). Emergence of Plasmid-Mediated Colistin Resistance Mechanism MCR-1 in Animals and Human Beings in China: A Microbiological and Molecular Biological Study. Lancet Infect. Dis..

[50] Chin C.-Y., Gregg K.A., Napier B.A., Ernst R.K., Weiss D.S. (2015). A PmrB-Regulated Deacetylase Required for Lipid A Modification and Polymyxin Resistance in *Acinetobacter baumannii*. Antimicrob. Agents Chemother..

[51] Knopp M., Babina A.M., Gudmundsdóttir J.S., Douglass M.V., Trent M.S., Andersson D.I. (2021). A Novel Type of Colistin Resistance Genes Selected from Random Sequence Space. PLoS Genet..

[52] Quesada A., Porrero M.C., Téllez S., Palomo G., García M., Domínguez L. (2015). Polymorphism of Genes Encoding PmrAB in Colistin-Resistant Strains of *Escherichia coli* and *Salmonella enterica* Isolated from Poultry and Swine. J. Antimicrob. Chemother..

[53] Sun S., Negrea A., Rhen M., Andersson D.I. (2009). Genetic Analysis of Colistin Resistance in *Salmonella enterica* Serovar Typhimurium. Antimicrob. Agents Chemother..

[54] Luther A., Urfer M., Zahn M., Müller M., Wang S.-Y., Mondal M., Vitale A., Hartmann J.-B., Sharpe T., Monte F.L. (2019). Chimeric Peptidomimetic Antibiotics against Gram-Negative Bacteria. Nature.

[55] Vetterli S.U., Zerbe K., Müller M., Urfer M., Mondal M., Wang S.-Y., Moehle K., Zerbe O., Vitale A., Pessi G. (2018). Thanatin Targets the Intermembrane Protein Complex Required for Lipopolysaccharide Transport in *Escherichia coli*. Sci. Adv..

[56] Elliott A.G., Huang J.X., Neve S., Zuegg J., Edwards I.A., Cain A.K., Boinett C.J., Barquist L., Lundberg C.V., Steen J. (2020). An Amphipathic Peptide with Antibiotic Activity against Multidrug-Resistant Gram-Negative Bacteria. Nat. Commun..

[57] Krenev I.A., Panteleev P.V., Umnyakova E.S., Gorbunov N.P., Kostevich V.A., Balandin S.V., Ovchinnikova T.V., Aleshina G.M., Berlov M.N. (2022). In Vitro Modulation of Complement Activation by Therapeutically Prospective Analogues of the Marine Polychaeta Arenicin Peptides. Mar. Drugs.

[58] Panteleev P.V., Ovchinnikova T.V. (2017). Improved Strategy for Recombinant Production and Purification of Antimicrobial Peptide Tachyplesin I and Its Analogs with High Cell Selectivity. Biotechnol. Appl. Biochem..

[59] Panteleev P.V., Bolosov I.A., Khokhlova V.A., Dhanda G., Balandin S.V., Haldar J., Ovchinnikova T.V. (2022). Analysis of Antibacterial Action of Mammalian Host-Defense Cathelicidins and Induction of Resistance to Them in MβL-Producing *Pseudomonas aeruginosa*. Bull. Exp. Biol. Med..

[60] Panteleev P.V., Balandin S.V., Ovchinnikova T.V. (2017). Effect of Arenicins and Other β-Hairpin Antimicrobial Peptides on *Pseudomonas aeruginosa* PAO1 Biofilms. Pharm. Chem. J..

