# Supplementary material for: Genomic Insights into Bacterial Resistance to Proline-Rich Antimicrobial Peptide Bac7
Source: Membranes (Basel). 2023 Apr 17;13(4):438. doi: 10.3390/membranes13040438 (PMC10145973; doi:10.3390/membranes13040438)
Supplement: Supplementary file 1 [file membranes-13-00438-s001.zip › membranes-2305651-supplementary.pdf]

Supplementary Materials

Genomic insights into bacterial resistance to proline-rich antimicrobial peptide Bac7

Pavel V. Panteleev, Victoria N. Safronova, Roman N. Kruglikov, Ilia A. Bolosov, Tatiana V. Ovchinnikova*

M.M. Shemyakin & Yu.A. Ovchinnikov Institute of Bioorganic Chemistry, the Russian Academy of Sciences, Miklukho-Maklaya str., 16/10, 117997 Moscow, Russia; victoria.saf@ibch.ru (V.N.S.); kruglikov1911@mail.ru (R.N.K.); bolosov@ibch.ru (I.A.B.); contraton@mail.ru (I.V.B.); ovch@ibch.ru (T.V.O.)

* Correspondence: ibch@inbox.ru; Tel.: +7-495-335-09-00

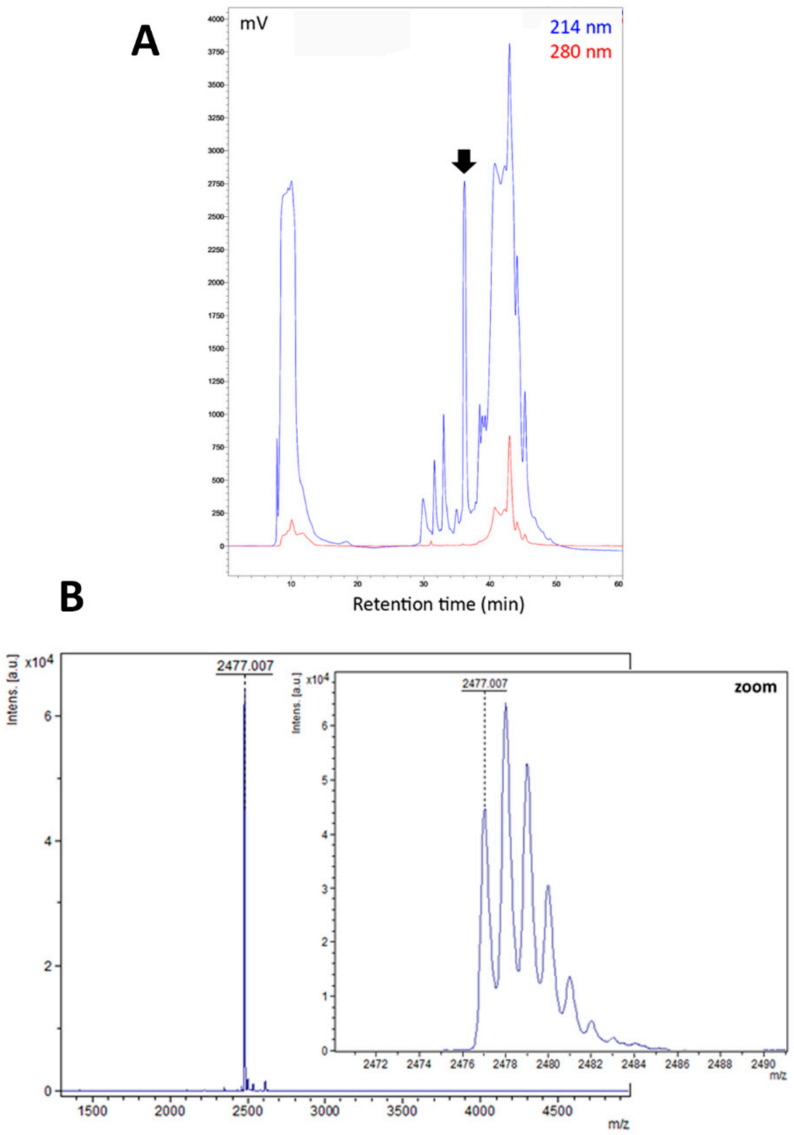

Figure S1. Purification of the recombinant pexiganan. **(A)** Reverse-phase high-performance liquid chromatography (RP-HPLC) purification of the recombinant pexiganan. The fraction of the target peptide is marked with an arrow. **(B)** MALDI-TOF mass spectrometry analysis of the recombinant pexiganan. The experimental $[M + H]^+$ monoisotopic mass is presented in the picture.

Antimicrobial	Class	WGS-predicted phenotype	Genetic background
amikacin	aminoglycoside	No resistance	
tigecycline	tetracycline	No resistance	
tobramycin	aminoglycoside	No resistance	
cefepime	beta-lactam	Resistant	blaCTX-M-15 (blaCTX-M-15_AY044436)
chloramphenicol	amphenicol	No resistance	
piperacillin+tazobactam	beta-lactam	No resistance	
cefoxitin	beta-lactam	No resistance	
ampicillin	beta-lactam	Resistant	blaCTX-M-15 (blaCTX-M-15_AY044436)
ampicillin+clavulanic acid	beta-lactam	No resistance	
cefotaxime	beta-lactam	Resistant	blaCTX-M-15 (blaCTX-M-15_AY044436)
ciprofloxacin	quinolone	Resistant	gyrA (p.S83L)
colistin	polymyxin	No resistance	
sulfamethoxazole	folate pathway antagonist	Resistant	sul1 (sul1_U12338)
imipenem	beta-lactam	No resistance	
trimethoprim	folate pathway antagonist	No resistance	
nalidixic acid	quinolone	Resistant	gyrA (p.S83L), gyrA (p.D87N)
ertapenem	beta-lactam	No resistance	
tetracycline	tetracycline	No resistance	
fosfomycin	fosfomycin	No resistance	
ceftazidime	beta-lactam	Resistant	blaCTX-M-15 (blaCTX-M-15_AY044436)
temocillin	beta-lactam	No resistance	
gentamicin	aminoglycoside	No resistance	
meropenem	beta-lactam	No resistance	
azithromycin	macrolide	Resistant	mph(A) (mph(A)_D16251)

Figure S2. Identification of antibiotic resistance genes in *E. coli* MDR 1057 strain performed using ResFinder 4.1 (https://cge.food.dtu.dk/services/ResFinder/).

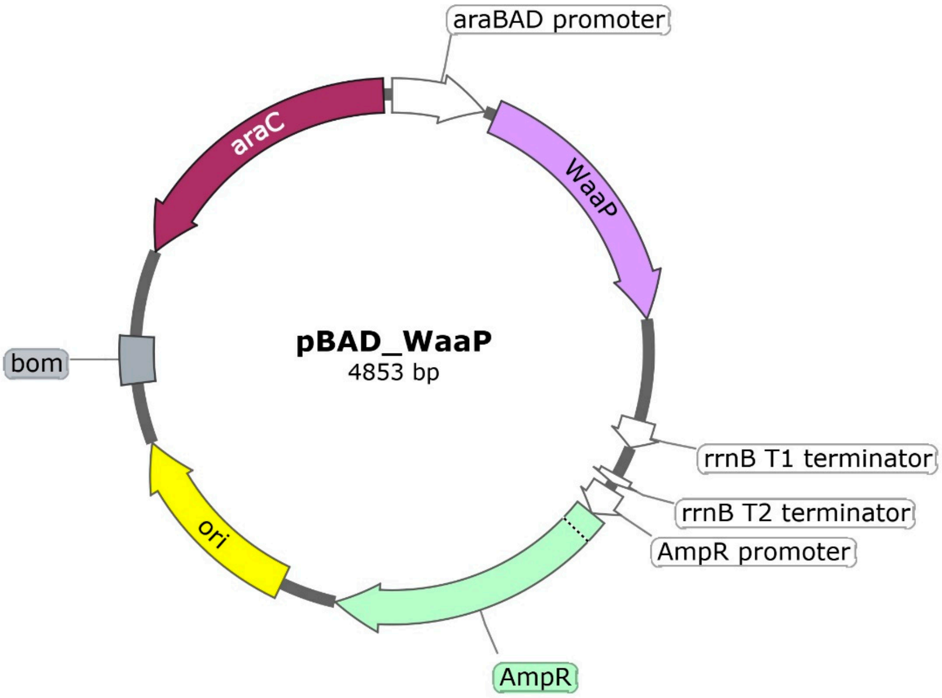

Figure S3. pBAD-based plasmid vector for the expression of *waaP* under the tightly regulated arabinose promoter. Map was visualized with SnapGene software.

```
…cggcgcatgcgctggggctgacggcggtgatcagttcttccattgagtcgagcttaggtctaatgcaactggcgcg
gattgctgcctggttaacgccagacaccattccgggactggacacgctggatctgatgcaggtgcagcaggttcgtc
gctggccggggagtccactgccgttagtggacgttgatgcactggagcggctgttatgatcttctctgactggccgt
ggcgtcactggcggcaagtgcggggagaagccatcgccttacgtcttaatgacgagcaactcaactggcgcgagctt
tgtgctcgcgtcgatgaattagcctccagctttgcggcgcagggtgtggttgcgggcagcggcgtgatgttgcgggc
gtggaatacgccgcaaacgctgctggcctggctggcgttactgcaatgcggggcgcgggtgttgcccgtgaaccctc
agctgccgcaaccgctgcttgaggaattgctgcccaatcttacgctgcaatttgcgctggtgccggaaggggaaaac
acgtttccggcgttaacgtcgctgcatattcagcaggttgaaggcgtacatgccgtggcgtggcagccgacgcgtct
gtgctcaatgacgttaacctctggttccactggcttgccgaaagccgctgttcatacttatcaggctcatcttgcca
gcgccgaaggggtgttatcgctgattccgtttggcgatcacgatgactggttgctctctttaccgctgtttcacgtc
tccggtcagggaattatgtggcgctggttatacgctggtgcgcggatgacggtacgtgataaacagccattggagca
aatgctggcaggctgtacgcacgcttcactggtgccaacacaactctggcgtttgctggttaaccgtagttccgttt
ccctgaaagcggttttacttggcggcgcggctatcccggtcgagttgacggaacaggcgcgcgagcaggggattcgg
tgcttttgcggctatggtctgaccgagtttgcctccacggtgtgtgcgaaagaagccgacggtctggcagacgtcgg
ttcgccgctgccgggtcgggaagtgaaaatcgtaaacgacgaagtgtggctgcgggctgccagcatggcagaaggtt
actggcgtaacgggcaacgggttccactggttaatgacgaaggctggtacgccacgcgcgatcgcggtgagatgcat
aatggcaagctgaccattgtcggacgtttagacaatctgttcttcagtggtggcgaaggcattcagccagaagaagt
cgagcgcgttattgctgcacatcctgcggttttgcaggtgtttatcgttcccgttgctgacaaggagtttggtcatc
gaccggtagcggtggtggagtatgaccagcaaaccgttgatcttggcgaatgggtgaaagataagctggcccgtttt
caacaaccggtgcgctggctgactttaccgccggagctgaaaaacggcggtattaaaatttcacgtcaggcgttaaa
agagtgggtgcaacgccaggattaagaaatcattagtttcacttctcaattttgccattccattgccgttgataata
ggtttatctgttaatctgtaaattgatgtgaaaactcttatcaaacaggataatgcaatggaatggctggtcaaaaa
atcgtgctgcaataaacaaaataacagacatgtacttatgctctgcgatgctggcggcgcgataaaaatgatcgccg
aagtgaagagtgacttcgccgtgaaagtggggatttactctcgcctttgcagaatgcgctttactgtattaatcgt
gaaaagctgcacacggtgaaagttctttctgcaagctgttatagccccgatgagtgggaacggcagtgtaaagcggc
agggaaaactcagtaacgttgttttgttgtgggcaggaaaatcagcatcaacccgctcataatacaggctactccca
gtagtgctttcagcgaaaaagtcccttcccagccgggaagtaccattgaagcaatccataccagcacataactcata
ctgagcaaggcataggctttgcttagcgcaagtttatgtagtgttttataccaacaaaaaacggaaagcagataa…
```

Figure S4. A fragment of *E. coli* MDR 1057 genome sequence showing intergenic *menE-pmrD* region. The found SNP mutation is highlighted with yellow color. Start and termination codons in the corresponding genes are marked with green and red color, respectively.

Table S1. Oligonucleotide primers used in this study

Name	Sequence 5'→3'	Description
Pex-f	GC<u>AGATCT</u>CATATGGGGATCGGCAAATTCTTAAAGAAAGCGAAGAAGTTCGGGAAAGCC	Synthesis of DNA fragment encoding pexiganan followed by insertion into pET expression plasmid
Pex-r	GC<u>GAATTC</u>TTATTTCTTCAGAATTTTCACAAAGGCTTTCCCGAACTTCT	
Sbm-f	GTCGAAACAATTCTTATGGTCAG	Amplification of *sbmA* gene of *E. coli* (TA-cloning followed by Sanger sequencing)
Sbm-r	GCGAAGATAGAGGATTGACGCG	
Waa-f	GC<u>CCATGG</u>TTGAACTTAAAGAGCCGTTTG	Amplification of *waaP* gene of *E. coli* to insert into pBAD plasmid by NcoI/EcoRI sites (underlined)
Waa-r	CG<u>GAATTC</u>TTATAATCCTTTGCGTTGTGTTCGC	

Table S2. Amino acid sequences and molecular masses of AMPs used in this study

Peptide	Origin	Sequence	Molecular mass, Da		Source
			Calculated [M+H]+ value[1]	Measured value[2]	
Bac7$_{1\text{-}22}$	Rec	RRIRPRPPRLPRPRPRPLPFPR	2783.72	2783.89	[1]
PR-39$_{1\text{-}22}$	Rec	RRRPRPPYLPRPRPPPFFPPRL	2765.61	2765.35	[1]
mini-ChBac7.5Nα	Rec	RRLRPRRPRLPRPRPRPRPRPR	2894.81	2894.05	[2]
VicBac	Synt	RRIRRPRLPRPRVPRPRIPPRIPRPVLPPPRVPFPRFPR*	4815.98	4815.70	[1]
AA139	Rec	GFCWYVCARRNGARVCYRRCN	2549.17	2550.07	[3]
Tachyplesin-1	Rec	KWCFRVCYRGICYRRCR	2264.08	2263.73	[4]
Protegrin-1	Rec	RGGRLCYCRRRFCVCVGR	2156.07	2156.01	[5]
Thanatin	Rec	GSKKPVPIIYCNRRTGKCQRL	2415.32	2415.36	[6]
Pexiganan	Rec	GIGKFLKKAKKFGKAFVKILKK	2476.61	2477.01	This study[3]
ChMAP-28	Rec	GRFKRFRKKLKRLWHKVGPFVGPILHY	3364.00	3364.22	[2]
Melittin	Synt	GIGAVLKVLTTGLPALISWIKRKRQQ	2846.74	2846.60	[1]
LL-37	Synt	LLGDFFRKSKEKIGKEFKRIVQRIKDFLRNLVPRTES	4491.58	4491.40	[1]

Rec – recombinant peptide

Synt – synthetic peptide

* *C*-terminal amidation

[1] according to Expasy Isotopident tool

[2] monoisotopic m/z were measured using MALDI-TOF MS

[3] final yield of pexiganan was 6.7 mg per liter of the culture medium.

Supplementary References:

1. Panteleev, P.V.; Safronova, V.N.; Kruglikov, R.N.; Bolosov, I.A.; Bogdanov, I.V.; Ovchinnikova, T.V. A Novel Proline-Rich Cathelicidin from the Alpaca Vicugna Pacos with Potency to Combat Antibiotic-Resistant Bacteria: Mechanism of Action and the Functional Role of the C-Terminal Region. *Membranes* **2022**, *12*, 515, doi:10.3390/membranes12050515.
2. Panteleev, P.V.; Bolosov, I.A.; Kalashnikov, A.À.; Kokryakov, V.N.; Shamova, O.V.; Emelianova, A.A.; Balandin, S.V.; Ovchinnikova, T.V. Combined Antibacterial Effects of Goat Cathelicidins With Different Mechanisms of Action. *Front. Microbiol.* **2018**, *9*, 2983, doi:10.3389/fmicb.2018.02983.
3. Krenev, I.A.; Panteleev, P.V.; Umnyakova, E.S.; Gorbunov, N.P.; Kostevich, V.A.; Balandin, S.V.; Ovchinnikova, T.V.; Aleshina, G.M.; Berlov, M.N. In Vitro Modulation of Complement Activation by Therapeutically Prospective Analogues of the Marine Polychaeta Arenicin Peptides. *Mar. Drugs* **2022**, *20*, 612, doi:10.3390/md20100612.
4. Panteleev, P.V.; Ovchinnikova, T.V. Improved Strategy for Recombinant Production and Purification of Antimicrobial Peptide Tachyplesin I and Its Analogs with High Cell Selectivity. *Biotechnol. Appl. Biochem.* **2017**, *64*, 35–42, doi:10.1002/bab.1456.
5. Panteleev, P.V.; Bolosov, I.A.; Khokhlova, V.A.; Dhanda, G.; Balandin, S.V.; Haldar, J.; Ovchinnikova, T.V. Analysis of Antibacterial Action of Mammalian Host-Defense Cathelicidins and Induction of Resistance to Them in MβL-Producing Pseudomonas Aeruginosa. *Bull. Exp. Biol. Med.* **2022**, *172*, 447–452, doi:10.1007/s10517-022-05411-8.
6. Panteleev, P.V.; Balandin, S.V.; Ovchinnikova, T.V. Effect of Arenicins and Other β-Hairpin Antimicrobial Peptides on Pseudomonas Aeruginosa PAO1 Biofilms. *Pharm. Chem. J.* **2017**, *50*, 715–720, doi:10.1007/s11094-017-1518-2.
